# The Unexpected Pitter Patter: New-Onset Atrial Fibrillation in Pregnancy

**DOI:** 10.1155/2015/318645

**Published:** 2015-04-15

**Authors:** Sarah White, Janna Welch, Lawrence H. Brown

**Affiliations:** Dell School of Medicine, University of Texas at Austin, USA

## Abstract

*Background*. Atrial fibrillation is a relatively uncommon but dangerous complication of pregnancy. Emergency physicians must know how to treat both stable and unstable tachycardias in late pregnancy. In this case, a 40-year-old female with a cerclage due to incompetent cervix and previous preterm deliveries presents in new-onset atrial fibrillation. *Case Report*. A previously healthy 40-year-old African American G2 P1 female with a 23-week twin gestation complicated by an incompetent cervix requiring a cervical cerclage presented to the emergency department with intermittent palpitations and shortness of breath for the past two months. EMS noted the patient to have a tachydysrhythmia, atrial fibrillation with rapid ventricular response. She was placed on a diltiazem drip, which was titrated to 15 mg/hr without successful rate control. Her heart rate remained in the 130s and the rhythm continued to be atrial fibrillation with RVR. Digoxin was then added as a second agent, and discussions about the potential risks of cardioversion in pregnancy ensued. Fortunately, the patient converted to sinus rhythm before cardioversion became necessary. The digoxin was discontinued; the diltiazem was also discontinued after the patient subsequently developed hypotension. *“Why Should Emergency Physicians Be Aware of This?”* New-onset atrial fibrillation is rare in pregnancy but can increase the mortality and morbidity of the mother and fetus if not treated promptly.

## 1. Introduction

Atrial fibrillation is a relatively uncommon but dangerous complication of pregnancy. Emergency physicians must know how to treat both stable and unstable tachycardias in late pregnancy. In this case, a 40-year-old female with a cerclage due to incompetent cervix and previous preterm deliveries presents in new-onset atrial fibrillation.

## 2. Case Report

A previously healthy 40-year-old African American G2 P1 female with a 23-week twin gestation complicated by an incompetent cervix requiring a cervical cerclage presented to the emergency department with intermittent palpitations and shortness of breath for the past two months. The patient arrived via EMS complaining of persistent shortness of breath and palpitations for the past six hours. She was noted by EMS to have a tachydysrhythmia, atrial fibrillation with rapid ventricular response (RVR), and received two doses of diltiazem en route: 20 mg and then another 25 mg without rate control.

Upon arrival, the patient's shortness of breath had resolved but she continued to have palpitations. The patient denied dehydration, alcohol or caffeine use, lack of sleep, recent illness, or any other precipitating factors. Her past medical history was significant only for a previous preterm delivery at 20 weeks due to an incompetent cervix. She denied any cardiomyopathies, prior arrhythmias, or other structural heart diseases.

On physical exam, the patient was afebrile, normotensive, with a heart rate in the 130s–140s, and a respiratory rate of 25–42. Abnormal physical exam findings included bilateral crackles in the lung bases and an irregularly irregular heartbeat. Fetal heart tones were observed in both fetuses.

Initial work-up included an EKG, CBC, CMP, UA with microscopy, chest X-ray, cardiac enzymes, D-Dimer, BNP, and thyroid studies. On EKG, rate was 142 and rhythm was atrial fibrillation with rapid ventricular response. The chest radiograph revealed cardiac hypertrophy and right greater than left perihilar opacities suggestive of pulmonary edema. Laboratory examination revealed mild leukocytosis and mild elevation of D-Dimer, with the remainder of the results within normal limits.

The patient was placed on a diltiazem drip, which was titrated to 15 mg/hr without successful rate control. Her heart rate remained in the 130s and the rhythm continued to be atrial fibrillation with RVR. Digoxin was then added as a second agent, and discussions about the potential risks of cardioversion in pregnancy ensued. Fortunately, the patient converted to sinus rhythm before cardioversion became necessary. The digoxin was discontinued; the diltiazem was also discontinued after the patient subsequently developed hypotension.

The patient was admitted to hospital and was followed closely in an intermediate care unit by cardiology and OB/GYN. An ECHO was completed during her hospital admission that showed moderate left ventricular hypertrophy (LVH), and on further laboratory examination she appeared to likely have gestational diabetes. Due to her LVH, flecainide was not recommended, and due to her pregnancy it was decided that aspirin would be used for anticoagulation. The patient was discharged with follow-up with cardiology, high-risk obstetrics, and maternal fetal monitoring for the remainder of her pregnancy.

## 3. Discussion

There are many physiologic changes that occur during pregnancy, including increased demands on the cardiovascular system. Physiologic changes in the cardiovascular system include peripheral vasodilation resulting in decreased systemic vascular resistance, requiring increased cardiac output. This increase in cardiac output is accomplished by an increase in ventricular end-diastolic volume, wall mass, and contractility, which creates an increase in stroke volume and heart rate. Due to these alterations, pregnant women are placed at higher risk for developing comorbidities such as cardiac arrhythmias that range from benign to life threatening [4, 5].

While most cases of atrial fibrillation in pregnancy are the result of an underlying cardiac arrhythmia or structural abnormality, there is a very small percentage of women who develop new-onset “lone” atrial fibrillation during their pregnancy. While these cases are known in the OB/GYN literature, their presentation and management in the emergency department have not previously been described in emergency medicine literature.

In evaluating new-onset “lone” atrial fibrillation in the pregnant woman, it is important to discern the etiology of the abnormality. The initial work-up should attempt to rule out cardiac conduction and structural abnormalities, as well as extracardiac etiologies such as pulmonary embolus, hyperthyroidism, electrolyte disturbances, and pharmacologic effects. This assessment should include an EKG, echocardiogram, serum electrolytes, thyroid studies, and urine drug screen. The EKG should reveal the diagnosis of atrial fibrillation as well as any other conduction abnormalities once the rate is controlled, and an echocardiogram will be able to evaluate any structural abnormalities that would predispose the patient to developing an arrhythmia. A thorough evaluation of medications as well as illicit drug use should be completed, as many pharmacologic agents are arrhythmogenic. If clinically indicated, an evaluation for pulmonary embolus should be completed using laboratory and radiologic evidence and therapy should be initiated [6].

Emergency department treatment is divided between pharmacologic and nonpharmacologic interventions for rate and rhythm control. The initial goal should be to control the rate; however, in order to maintain consistent blood-flow to the patient's end organs including the placenta, sinus rhythm should be restored as soon as possible. The European Society of Cardiology established guidelines in 2011 for management of cardiovascular diseases in pregnancy. Beta-blockers are the first choice for rate control of atrial fibrillation in pregnancy, followed by calcium-channel blockers. They recommend digoxin as well, with the caveat that digoxin blood concentrations are unreliable in pregnancy due to interference with immunoreactive serum components [7].

When choosing therapeutic interventions, adverse events to both mother and fetus must be taken into consideration, as many antiarrhythmic drugs are teratogenic. The FDA classifies the risk and benefits of medications during pregnancy as follows:

 A: controlled studies showed no risk in the fetus in any trimester of pregnancy.
 B: no evidence of risk in humans. The chance of fetal harm is remote.
 C: the risk cannot be ruled out. There are no well-controlled clinical studies, and animal studies show risk to the fetus. Fetal damage is likely if the drug is administered during pregnancy, but the potential benefits could exceed the potential risk.
 D: sure evidence of risk. However, the potential benefits of use of the drug may outweigh the potential risk.
 X: contraindicated in pregnancy. Studies in animals or humans showed certain evidence or risk of fetal abnormality and clearly outweigh any benefit to the patient [8].

Table 1 shows the FDA classification and safety profile for the pharmacologic agents used in the emergency department treatment of atrial fibrillation, modified from Oishi and Xing [9].

Emergency physicians frequently encounter patients with atrial fibrillation that does not respond to pharmacologic intervention, and cardioversion is a common emergency department intervention. Pregnant women with unresponsive, new-onset “lone” atrial fibrillation are rare, and emergency physicians are likely not as conversant with the risks and benefits of cardioversion in this patient group. In hemodynamically unstable patients, in patients who are not responding to pharmacologic therapy, or whenever the risk of ongoing atrial fibrillation is considered high for the mother or the fetus cardioversion up to 400 J can be performed safely at all stages of pregnancy [6]. It is a Class I recommendation with a C level of evidence [9]. That is, risk to the fetus cannot be ruled out. While maternal cardioversion is unlikely to have significant effects on the fetus due to a high threshold for arrhythmogenesis and the minimal amount of current that reaches the uterus [10], continuous fetal monitoring should be utilized during the procedure, and cardioversion should be performed in a facility with the ability to perform an emergency C-section if needed. Previous reports note fetal arrhythmia developing as bradycardia or loss of variability after maternal cardioversion; both of which are indications for C-section [11].

In cases of atrial fibrillation occurring for longer than 48 hours, a transesophageal ECHO should be completed to evaluate atrial thrombus, and anticoagulation should be initiated prior to cardioversion [6, 8]. Vitamin K agonists, however, are teratogenic and therefore unfractionated or low molecular weight heparin should be used during the first trimester. During the third trimester, frequent laboratory testing is recommended to maintain appropriate levels of anticoagulation [8, 12].

### 3.1. “Why Should Emergency Physicians Be Aware of This?”

New-onset atrial fibrillation is rare in pregnancy but can increase the mortality and morbidity of the mother and fetus if not treated promptly. In the case of an unstable patient, cardioversion should be completed as quickly as possible. In a stable patient, effort should be made to determine the etiology of the arrhythmia, and treatment should follow accordingly. In the emergency department pharmacological intervention should be targeted toward rate control with Beta-blockers, calcium-channel blockers, or digoxin. In the case of our patient, management with diltiazem and digoxin was within European Society of Cardiology guidelines. Strict adherence to guidelines would have included beta-blocker as a first line medication. Once the rate is controlled, the provider should attempt to restore sinus rhythm chemically or electrically. In this case, the patient may require anticoagulation prior to cardioversion, with unfractionated or low molecular weight heparin. A high-risk obstetrician for the remainder of their pregnancy should follow these patients, and continued monitoring and treatment of her arrhythmia should be performed in conjunction with a cardiologist.

## Figures and Tables

**Table 1:** 

Drug	Rate versus rhythm control	Class of recommendation/level of evidence	FDA category	Dosage	Adverse effects
Beta-blockers	Rate	Class IIa/C	C		Pregnancy: born small for gestational age, preterm birth, and perinatal mortality [1]. General: bradycardia, hypotension, AV block, bronchospasm
Esmolol				Loading: 0.5 mg/kg over 1 min. Maintenance: 0.06–0.2 mg/k/min	
Metoprolol				2.5–5 mg bolus over 2 min, up to 3 doses	
Propranolol				0.15 mg/kg	
Nondihydropyridine calcium channel blockers	Rate	Class IIa/C	C		Pregnancy: increased risk of neonatal seizures, jaundice, and hematologic disorders [2]. General: hypotension, heart failure
Diltiazem				Loading: 0.25 mg/kg/dose over 2 min; may give a second dose at 0.35 mg/kg/dose. Maintenance: 5–15 mg/kg for <24 hr.	
Verapamil				0.075–0.15 mg/kg over 2 min	

Cardiac glycoside					
Digoxin	Rate	Class IIb/C	C	Loading: 0.25 mg IV every 2 h, up to 1.5 mg. Maintenance 0.125–0.375 mg daily IV or orally	Pregnancy: digitalis toxicity may cause fetal demise [3]. General: digitalis toxicity, heart block

Class I C antiarrhythmic agent					
Flecainide	Rhythm	Class IIb/C	C		General: heart block, ventricular arrhythmias, and heart failure

Class III antiarrhythmic agent					
Ibutilide	Rhythm	Class IIb/C	C	1 mg IV; may repeat dose if no response after 10 min	General: bradycardia, AV block, Torsades

Adjunctive therapies					
Magnesium		NA	B	2 g over 15 min	General: respiratory depression
